# Ketone ester–enriched diet ameliorates motor and dopamine release deficits in MitoPark mice

**DOI:** 10.1111/ejn.16601

**Published:** 2024-11-11

**Authors:** Vikrant R. Mahajan, Jacob A. Nadel, M. Todd King, Robert J. Pawlosky, Margaret I. Davis, Richard L. Veech, David M. Lovinger, Armando G. Salinas

**Affiliations:** ^1^ Laboratory for Integrative Neuroscience National Institute on Alcohol Abuse and Alcoholism, National Institutes of Health Rockville Maryland USA; ^2^ Laboratory for Metabolic Control National Institute on Alcohol Abuse and Alcoholism, National Institutes of Health Rockville Maryland USA; ^3^ Department of Pharmacology, Toxicology & Neuroscience Louisiana State University Health Sciences Center – Shreveport Shreveport Louisiana USA

**Keywords:** dopamine neurons, ketosis, neuroprotection, Parkinson's disease, striatum

## Abstract

Parkinson's disease (PD) is a progressive, neurodegenerative disease characterized by motor dysfunction and dopamine deficits. The MitoPark (MP) mouse model of PD recapitulates several facets of Parkinson's disease, including gradual development of motor deficits, which enables the study of potential therapeutic interventions. One therapeutic strategy involves decreasing the mitochondrial metabolic load by inducing ketosis and providing an alternative energy source for neurons, leading to decreased neuronal oxidative stress. Thus, we hypothesized that administration of a ketone ester–enriched diet (KEED) would improve motor and dopamine release deficits in MP mice. Motor function (rotarod and open field tests), dopamine release (fast‐scan cyclic voltammetry), tissue dopamine levels (gas chromatography–mass spectrometry) and dopamine neurons and axons (immunofluorescence) were assessed in MP, and control mice fed either the standard or a KEED. When started on the ketone diet before motor dysfunction onset, MP mice had improved motor function relative to standard diet (SD) MP mice. While the KEED did not preserve dopamine neurons or striatal dopamine axons, dopamine release in ketone diet MP mice was greater than SD MP mice but less than control mice. In a follow‐up experiment, we began the ketone diet after motor dysfunction onset and observed a modest preservation of motor function in ketone diet MP mice relative to SD MP mice. The improvement in motor dysfunction indicates that a KEED or ketone supplement may have a beneficial effect on delaying motor deficit progression in Parkinson's disease.

## Graphical abstract

MitoPark mice, a model of Parkinson's disease, fed a ketone ester–enriched diet had moderate improvements in locomotor performance on open field and rotarod behavioural tasks. This behavioural improvement was accompanied by increases in dorsal striatal dopamine release. However, the ketone diet did not preserve dopamine neurons or putative striatal dopamine axons.

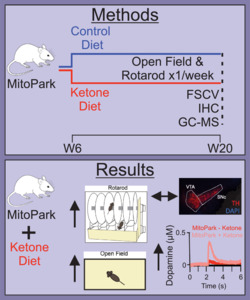

**Abbreviations**
6‐OHDA: 6‐hydroxydopamine
BH2: dihydrobiopterin
BH4: tetrahydrobiopterin
C − k: control mouse/mice without ketone ester–enriched diet
C + k: control mouse/mice with ketone ester–enriched diet
D‐BHB: D‐β‐Hydroxybutyrate
GC–MS: gas chromatography–mass spectrometry
HDAC: histone deacetylase
KEED: ketone ester–enriched diet
MPTP: 1‐methyl‐4‐phenyl‐1,2,3,6‐tetrahydropyridine
MP: MitoPark
MP − k: MitoPark mouse/mice without ketone ester–enriched diet
MP + k: MitoPark mouse/mice with ketone ester–enriched diet
PD: Parkinson's disease
ROS: reactive oxygen species
SD: standard diet
SNc: Substantia Nigra compacta
TCA: tricarboxylic acid
TH: tyrosine hydroxylase
VMAT2: vesicular monoamine transporter 2

## 1 INTRODUCTION

Parkinson's disease (PD) is a progressive, neurodegenerative disease characterized by hypokinesia, resting tremor and dystonia (Sveinbjornsdottir, 2016). It is the second most common neurodegenerative disease with a 1% prevalence in the population over 60 years old (Tysnes & Storstein, 2017). The underlying pathology of PD involves a progressive loss of dopaminergic neurons in the substantia nigra pars compacta (SNc) (Sveinbjornsdottir, 2016); however, the exact mechanisms underlying this loss remain elusive. Converging lines of evidence suggest a central role for mitochondrial dysfunction in PD aetiology (Park et al., 2018). Indeed, toxins targeting mitochondrial respiration produce selective vulnerability in SNc dopamine neurons (Przedborski et al., 2004; Sherer et al., 2003) and PD‐like behavioural disturbances. Additionally, dopamine neurons from PD patients were found to have more mitochondrial DNA deletions than age‐matched controls (Bender et al., 2006). Specifically, mitochondrial dysfunction is hypothesized to increase oxidative stress (Henchcliffe & Beal, 2008). The MitoPark (MP) model is a mouse model of PD in which the mitochondrial transcription factor TFAM A is selectively knocked out of dopamine neurons, resulting in a progressive dopaminergic neuron degeneration and a gradual onset of PD‐like motor deficits (Ekstrand et al., 2007). This model also recapitulates several important aspects of PD pathophysiology, such as the gradual development of motor deficits in adulthood, responsiveness to L‐DOPA and protein inclusions (Galter et al., 2010; Ghaisas et al., 2019). Recent work has attributed degeneration in MP mice to impaired antioxidant defense, which is itself due to mitochondrial dysfunction and calcium dishomeostasis (Ricke et al., 2020). Thus, the MP model is suitable not only to capture the hallmark progressive pathophysiology of PD but also to study the effects of potential therapeutic interventions.

In PD, L‐DOPA provides effective temporary motor symptom relief but does not address the underlying pathology of the disorder. Furthermore, neither L‐DOPA nor other dopamine replacement therapies help to preserve dopamine neuron function. The high‐fat/low‐carbohydrate (ketogenic) diet has been examined recently for its potential application in ameliorating neurological disease pathologies by inducing ketosis (Lambrechts et al., 2017; Phillips et al., 2018, 2021). Ketone bodies, including D‐β‐Hydroxybutyrate (D‐BHB), can supply peripheral tissues (e.g. muscle or cardiac tissues) with an energy supply when glucose levels are insufficient (Newman & Verdin, 2017). D‐BHB has also been shown to have a beneficial effect in some kidney disease models (Chang et al., 2021), colitis (Abdelhady et al., 2023; Huang et al., 2022), inflammation and pain (Ruskin et al., 2009). Exogenous oral administration of D‐BHB, in the form of a ketone ester–enriched diet (KEED), has been shown to ameliorate symptoms in rodent models of Alzheimer's (Pawlosky et al., 2020), metastatic cancer (Poff et al., 2014), diabetes (Sato et al., 1995) and epilepsy (Ciarlone et al., 2016; Viggiano et al., 2015). Furthermore, the bioenergetics of D‐BHB gives a mechanism of action for sequestering and quenching of reactive oxygen species (Boison et al., 2016). Given the aforementioned roles of mitochondrial dysfunction and oxidative stress in PD, we hypothesized that the KEED would alleviate PD symptoms in MP mice, potentially by reducing dopamine neuron metabolic load and/or increasing antioxidant availability.

Thus, we utilized the MP mouse model to assess the therapeutic potential of a KEED to curtail PD‐like symptoms. Specifically, we assessed the progressive loss of motor function under normal (open field) and challenging (rotarod) conditions in MP and control mice fed a standard diet (SD) or a KEED. In our first experiment, we began mice on the KEED before symptom onset and observed partial preservation of motor function in MP mice fed the KEED. This was paralleled by a modest preservation in dorsal striatal dopamine release measured with fast‐scan cyclic voltammetry (FSCV). Interestingly, this preservation of striatal dopamine release was not correlated with preserved dopamine neurons or dorsal striatal dopamine axon density in MP mice fed the KEED. Similarly, the KEED did not increase or preserve tonic dopamine (or its primary metabolite DOPAC) levels in dorsal striatum of MP mice. In a follow up experiment, we began feeding mice with the KEED after the onset of motor dysfunction and observed a more modest rescue of locomotor activity in the rotarod, but not open field, assay. Altogether, our results suggest that a KEED may have a beneficial effect on the progression of PD motor symptoms.

## 2 MATERIALS AND METHODS

For full details, see Supplemental Materials.

### 2.1 Animals

All procedures were performed in compliance with the National Institutes of Health Care and Use of Animals guidelines and approved by the Institutional Animal Care and Use Committee of the National Institute on Alcohol Abuse and Alcoholism. MP, and control mice were generated as previously described (Ekstrand et al., 2007; Galter et al., 2010; Good et al., 2011). Mice were housed up to four per cage on a 12‐h light/dark cycle in a temperature‐ and humidity‐controlled room with ad libitum access to food and water.

### 2.2 KEED

**FIGURE 1:**
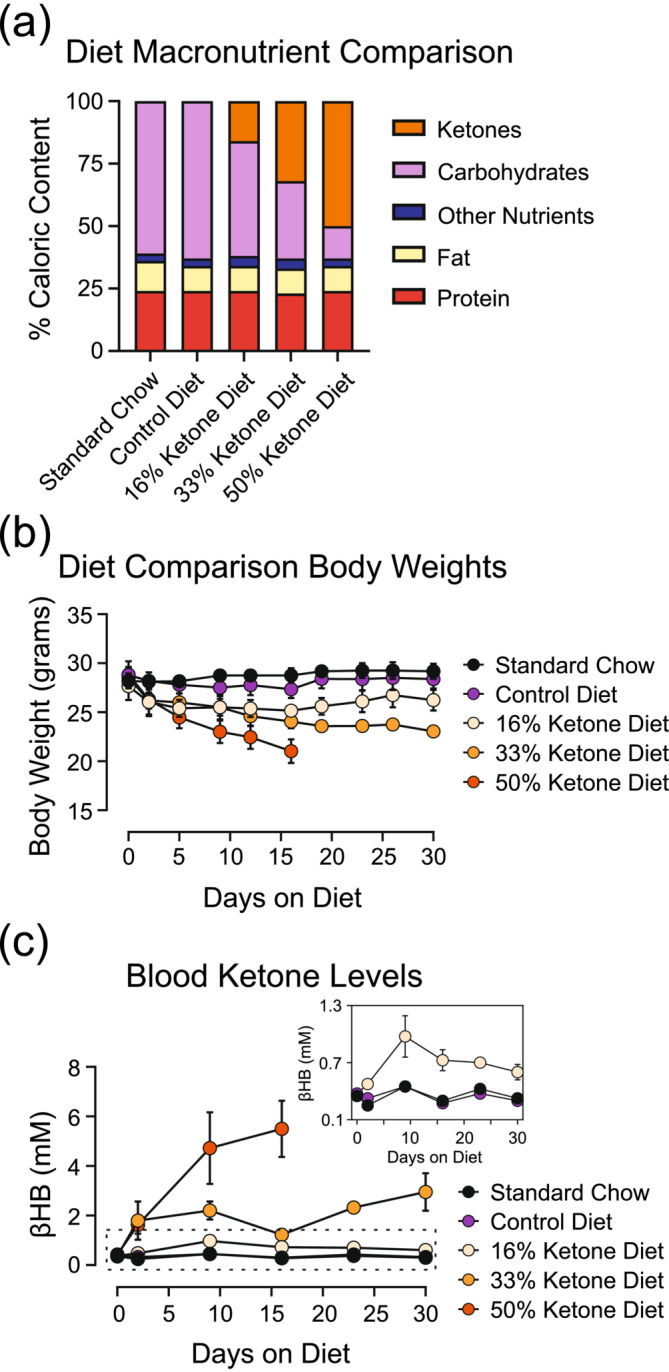
Comparison of ketone ester–enriched diets (KEEDs). (a) Macronutrient composition of tested KEEDs. Mice were fed each KEED for 30 days with body weights (b) and blood ketone levels (c) assessed periodically. *n* = 4 mice per diet group.

The KEED has been described previously (Kashiwaya et al., 2013). Briefly, the ketone ester ((R)‐3‐hydroxybutyl (R)‐3‐hydroxybutanoate) was produced in house and sent to Dyets, Inc. to prepare the diet. The KEED is based on the standard rodent diet but modified to include a ketone ester at the caloric expense of carbohydrates (Figure 1a). We tested diets with 16%, 33% and 50% of calories from ketone esters and chose the 16% KEED for use in subsequent experiments.

### 2.3 Blood ketone levels and diet testing

Male C57BL/6J mice (Jackson Laboratory, strain 000664) were used to determine achievable blood ketone levels for each diet: SD, control diet, 16% KEED, 33% KEED, and 50% KEED. Tail vein blood samples were collected at 0, 2, 9, 16, 23, and 30 days after starting the KEED. β‐Hydroxybutyrate levels were measured using Precision‐Xtra blood ketone test strips and a Precision‐Xtra meter (Abbott Labs, Abbott Park, IL, USA). Due to the circadian fluctuation of ketone levels (De Gasquet et al., 1977; Tognini et al., 2017), blood samples were obtained at the same point in the light cycle for each experiment (8–9 h into the light cycle).

### 2.4 Accelerating rotarod and open field

At 4 weeks of age, male and female pups were assigned to the SD or 16% KEED groups. Beginning at 6 weeks of age, MP and control mice fed either the SD or KEED were run in an open field task to assess spontaneous movement. Each week, mice were placed in large cages (42 cm × 22.5 cm × 25 cm) and video recorded for 1 h. The videos were processed in EthoVision (Noldus, Leesburg, VA), and average velocity for each mouse/session was calculated. On a different day in the same week, mice were run on an accelerating rotarod (EZRod, Omnitech Electronics or MedAssociates) to assess motor coordination and challenged movement. Because two rotarod apparatuses were needed to run all mice within a designated time period, mice were always assigned to the same lane and rotarod apparatus for the duration of testing. Mice were tested for 5 trials/day with the rotarod speed increasing from 4 to 40 rpm over 300 s. Trials were conducted every 10 min. The latency to fall from the rotarod was recorded for each trial. Individual trials were stopped, and latency was recorded if mice held onto the rod for two consecutive rotations or reached 40 rpm. Beginning at 8 weeks of age, a second group of mice was tested every other week on the rotarod and in the open field. These mice began the KEED at 14 weeks, after the onset of significant motor deficits. All behavioural testing was conducted between 12 and 3 PM local time (5–8 h into the light cycle).

### 2.5 FSCV

At 5, 10 or 20 weeks of age, mice were used for FSCV experiments to assess changes in dorsal striatal dopamine release. Briefly, 300‐μm‐thick brain slices containing the dorsal striatum were prepared as previously described (John & Jones, 2007). Dopamine release was electrically evoked using a range of stimulation intensities with a DS3 constant current stimulus generator (Digitimer NA, Ft. Lauderdale, FL) and a twisted, stainless steel stimulating electrode (P1 Technologies, Roanoke, VA). Carbon fibre electrodes were made as previously described (Salinas et al., 2016, 2021) and calibrated post hoc against a 1‐μM dopamine solution standard. Recording sites included both the dorsomedial and dorsolateral striatum. All KEED mice used for these experiments began the diet at 4 weeks.

### 2.6 Immunofluorescence

Mice were anesthetized and transcardially perfused with PBS followed by a 4% paraformaldehyde/PBS solution. Brains were postfixed overnight before sectioning into 50‐μm‐thick sections. The sections were washed 4 × 10 min in PBS‐T (0.2% Triton X‐100), then 2 × 10 min in 0.5% NaBH4 in PBS, then 4 × 10 min in PBS‐T before blocking for 2 h in 5% BSA in PBS‐T. Sections were then incubated overnight at 4°C in a primary antibody/0.5% BSA/PBS‐T solution of rabbit anti‐TH (1:1000, Invitrogen, Carlsbad, CA, 701949). Sections were then washed 6 × 10 min in PBS‐T before incubating in secondary antibody/0.5% BSA/PBS‐T solution of Alexa568 donkey antirabbit (1:2000) for 2 h. Sections were washed 6 × 10 min with PBS, mounted onto subbed slides, cover‐slipped with DAPI Fluoromount‐G (Electron Microscopy Sciences, Hatfield, PA) and imaged with a Zeiss AxioZoom microscope with ZEN software. Images were quantified using Fiji/Image J by a researcher blinded to the treatment and genotype of the mice. All KEED mice used for these experiments began the diet at 4 weeks.

### 2.7 Biochemistry

Dorsal striatal tissues from 20‐week‐old MP and control mice fed the SD or KEED were obtained and processed for dopamine and DOPAC levels with gas chromatography–mass spectrometry (GC–MS). Analytes were analyzed as its tertiary butyl dimethylsilyl ether‐ester derivatives using GC–MS in the electron impact mode and quantified using the ^2^H_3_‐acetate. Briefly, samples were homogenized and centrifuged, and the sample supernatant was collected and dried under nitrogen along with labelled internal standards before sylilation and processing on an Agilent 5973 quadrupole GC–MS (Agilent, Wilmington, DE). The ratios of the ions m/z 354/358 (dopamine retention time [RT], 13.1 min) and 384/389 (DOPAC, RT 12.85 min) were used to determine the quantity of each analyte.

### 2.8 Reagents

Unless otherwise indicated, reagents were obtained from Sigma‐Aldrich (St. Louis, MO). AlexaFluor‐conjugated secondary antibodies were obtained from Invitrogen (Carlsbad, CA).

### 2.9 Statistics

GraphPad Prism 8 was used for graphing and statistics. Where appropriate, we utilized one‐, two‐ and three‐way analyses of variance (ANOVAs) (with repeated measures and Geisser–Greenhouse corrections where necessary), as well as mixed‐effects analysis for data sets with missing values. When three‐way ANOVAs or mixed‐effects analyses had significant interactions, we performed two‐way analyses on the appropriate subgroups to better understand the differences between groups. Multiple comparisons were performed using Tukey's or Sidak's multiple comparisons tests.

## 3 RESULTS

For detailed results, see supplemental materials.

### 3.1 Comparison of KEEDs

We compared KEEDs with 16%, 33% and 50% of carbohydrate calories replaced by D‐BHB, alongside standard rodent chow and a control diet (Figure 1a) to maximize blood ketone levels with KEED palatability. We found that all KEEDs elevated blood ketone levels and that mice on each KEED initially lost body weight when switched to their respective diet (Figure 1b). Mice on the 16% KEED regained body weight by the third week on the diet, whereas mice on the 33% KEED did not recover their initial body weights by 30 days. Mice fed the 50% KEED were removed from the study at 16 days on the KEED due to severe weight loss. Given the weight loss in the 33% and 50% KEED groups, we chose the 16% KEED for subsequent experiments. Detailed statistical comparisons are available in the supplemental materials.

### 3.2 KEED ameliorates spontaneous locomotor deficits

**FIGURE 2:**
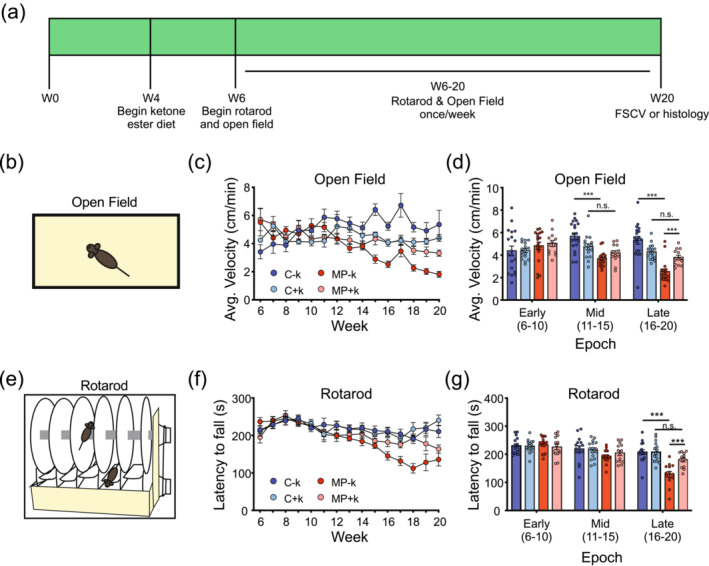
Presymptomatic KEED treatment ameliorated motor deficits in MitoPark mice. (a) Experimental timeline illustrating behavioral testing and diet onset. (b–d) Mice were tested in an open field to assess locomotion. MitoPark mice fed the SD exhibited decreased locomotion compared to MitoPark mice fed the 16% KEED (d). Mice were further tested in the rotarod apparatus (e). MitoPark mice fed the SD performed progressively worse (f) than KEED MitoPark and control mice (g). *n* = 10–14 mice per group. ***p < 0.001; n.s. *p* > 0.1. KEED, ketone ester–enriched diet; n.s., no significance; SD, standard diet.

From 6 to 20 weeks, mice were tested weekly in the open field (spontaneous locomotion) and on an accelerating rotarod (forced locomotion) (Figure 2). To decrease individual week‐to‐week performance variability, behavioural data was averaged into epochs: Early (weeks 6–10), Mid (11–15) and Late (16–20). In the open field (Figure 2c,d), mixed‐effects three‐factor mixed modeling analyses indicated significant effects of week (*F*_[2123]_ = 8.097, *p* = 0.0005) and genotype (*F*_[1,64]_ = 15.92, *p* = 0.0002), as well as significant week × genotype (*F*_[2123]_ = 20.76, *p* < 0.0001) and genotype × diet (*F*_[1,64]_ = 10.61, *p* = 0.0006) interactions, and a significant week × genotype × diet interaction (*F*_[2123]_ = 4.291, *p* = 0.0158). To investigate simple main effects, we performed two‐way mixed‐effects modeling across genotype and diet factors.

**Diet**: In control mice, there were significant effects of week (*F*_[1.457,48.82]_ = 4.591, *p* = 0.0242), and diet (*F*_[1,35]_ = 5.081, *p* = 0.0306). The significant effect of week was driven by an increased average velocity between Early and Mid epochs (*p* = 0.0162). The significant effect of diet was driven by the C − k mice having a significantly higher average velocity than C + k mice in the Late epoch (*p* = 0.0370). In MP mice, there were significant effects of week (*F*_[1.762,49.33]_ = 31.78, *p* < 0.0001) and diet (*F*_[1,29]_ = 5.872, *p* = 0.0219), and a trend towards a significant interaction (*F*_[2,56]_ = 3.134, *p* = 0.0513). The effect of week was driven by a decrease in average velocity across epochs (*p* < 0.002 for all). The effect of diet was driven by the MP + k mice moving significantly more than the MP − k mice in Late weeks (*p* = 0.0012). Furthermore, although MP − k mice significantly decreased average velocity across epochs (Early vs. Mid, *p* = 0.0129; Mid vs. Late, *p* = 0.0002; Early vs. Late, *p* < 0.0001), MP + k mice only showed significant deficits when comparing Early and Late epochs (Early vs. Mid, *p* = 0.1322, Mid vs. Late, *p* = 0.4888, Early vs. Late, *p* = 0.0004). Combined, these data suggest that MP mice fed a ketone diet develop delayed deficits in spontaneous locomotion relative to MP mice fed SD.

**Genotype**: In mice fed SD, there was a significant effect of week (*F*_[1.435,48.80]_ = 4.074, *p* < 0.0351) and genotype (*F*_[1,36]_ = 22.62, *p* < 0.0001) and a significant interaction (*F*_[2,68]_ = 16.70, *p* < 0.0001). C − k mice displayed slower average velocity on Early vs. Mid (*p* = 0.0475), but not Early vs. Late or Mid vs. Late epochs. In contrast, MP − k mice progressively declined across time points (Early vs. Mid, *p* = 0.0129; Mid vs. Late, *p* = 0.0002, Early vs. Late, *p* < 0.0001). Furthermore, MP − k mice had significant spontaneous movement deficits at both Mid (*p* < 0.0001) and Late (*p* < 0.0001) time points, compared to C − k. In KEED fed mice, there was a significant effect of week (*F*_[1.605,44.14]_ = 7.074, *p* = 0.0040) and a week × genotype interaction (*F*_[2,55]_ = 7.045, *p* = 0.0019). The effect of week was driven by the MP + k mice, whose velocity declined between Early and Late epochs (*p* = 0.0004). However, C + k and MP + k mice did not differ at any timepoint (*p* < 0.05). Overall, the presence of distinct effects across genotypes in SD but not KEED mice supports a beneficial effect of the KEED.

### 3.3 KEED ameliorates forced locomotion deficits on rotarod

To assess forced locomotion, motor coordination and balance, mice were run weekly on an accelerating rotarod (Figure 2f,g). Mixed‐effects three‐factor mixed modeling analyses revealed significant effects of week (*F*_[2113]_ = 59.53, *p* < 0.0001) and genotype (*F*_[1,59]_ = 12.35, *p* = 0.0009). Furthermore, there were significant week × genotype (*F*_[2113]_ = 18.64, *p* < 0.0001) and week × diet (*F*_[2113]_ = 0.5810, *p* = 0.0040) interactions, and a significant week × genotype × diet interaction (*F*_[2113]_ = 4.956, *p* = 0.0086). To investigate simple main effects, we performed two‐way mixed‐effects modeling, with mice split by genotype or diet factors.

**Diet**: In control mice, there was a significant effect of week (*F*_[1.863,54.96]_ = 5.745, *p* = 0.0064). Tukey's multiple comparisons tests indicated the significant effect of week was driven by a decreased latency to fall at the later weeks relative to early weeks (*p* = 0.0098) across diets. In MP mice, there was a significant effect of week (*F*_[1.895,51.16]_ = 75.88, *p* < 0.0001) and a significant week × diet interaction (*F*_[2,54]_ = 11.22, *p* < 0.0001), but no significant effect of diet alone (*F*_[1,29]_ = 3.3702, *p* = 0.0642). MP mice showed progressive decline across time (MP − k: all comparisons *p* < 0.0001; MP + k: Early vs. Mid, *p* = 0.0264, Mid vs. Late, *p* = 0.0166, Early vs. Late, *p* = 0.0007). However, MP + k mice had significantly longer latency to fall in Late weeks compared to the MP − k mice (*p* = 0.0014), indicating the KEED ameliorated forced locomotor deficits. Overall, these data indicate the KEED may improve forced locomotion in MP mice.

**Genotype**: In SD mice, there was a significant effect of week (*F*_[1.886, 55.65]_ = 50.10, *p* < 0.0001) and genotype (*F*_[1,31]_ = 12.18, *p* = 0.0015), and a significant interaction (*F*_[2,69]_ = 21.05, *p* < 0.0001). Multiple comparison tests indicated that C − k mice did not change performance across weeks, but in MP − k mice, latency to fall decreased across epochs (all *p* < 0.0001). Although latency to fall in MP − k mice declined across epochs, these mice only differed from the C − k mice at Late weeks (*p* < 0.0001). In KEED mice, there was a significant effect of week (*F*_[1.889,51.01]_ = 14.79, *p* < 0.0001) but no genotype or interaction effects. Tukey's multiple comparison tests indicate that the effect of week is driven by significant, or near‐significant, differences between all time points (Early vs. Mid, *p* = 0.0081; Mid vs. Late, *p* = 0.0580; Early vs. Late, *p* = 0.0003) regardless of diet. These analyses also support the ameliorative effect of the KEED on forced movement and motor coordination.

### 3.4 KEED ameliorates dopamine release deficits

**FIGURE 3:**
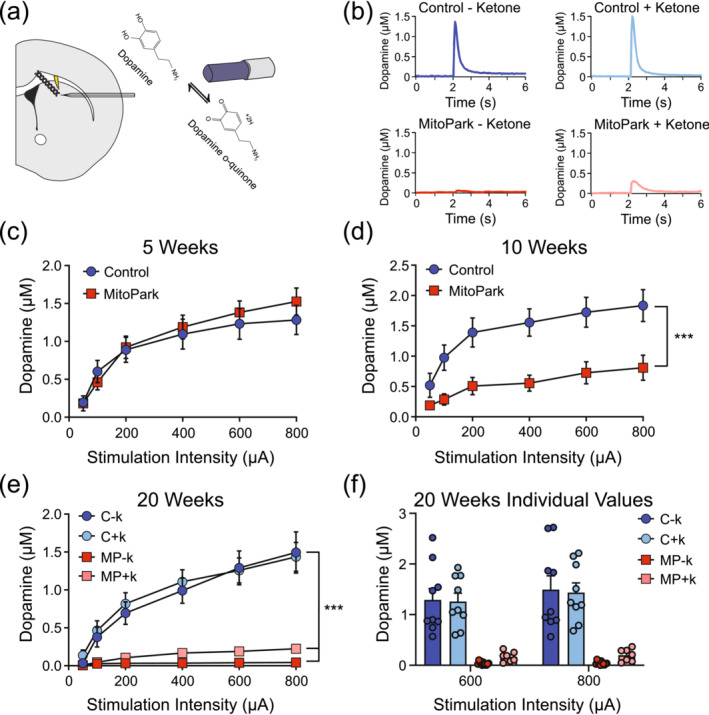
Progressive decrease in dopamine release in MitoPark mice is ameliorated by KEED. (a) Schematic for fast‐scan cyclic voltammetry experiments. (b) Representative dopamine release data for each group at 20 weeks. At 5 weeks (c), dopamine release did not differ between genotypes. (d) At 10 weeks, dopamine release was decreased in MitoPark mice. (e) At 20 weeks, dopamine release was decreased in MitoPark relative to control mice in SD groups. (f) MitoPark mice fed the KEED had greater dopamine release than MitoPark mice fed the SD. *n* = 7–12 slices per group from 5 to 7 mice per group. ***p < 0.001. KEED, ketone ester–enriched diet; SD, standard diet.

We assessed evoked dopamine release using FSCV in striatal slices from control and MP mice fed control diet at 5, 10 and 20 weeks of age (Figure 3). We also assessed dopamine release in 20‐week‐old mice fed the KEED. At 5 weeks, control and MP mice did not differ in evoked dopamine release (Figure 3c ). At 10 weeks, MP mice had lower dopamine release overall (significant main effect of genotype, *F*_(1,18)_ = 8.902, *p* = 0.0080; significant main effect of stimulation intensity, *F*_(1.185,21.33)_ = 22.44, *p* < 0.0001; significant interaction, *F*_(5,90)_ = 3.032, *p* = 0.0142) (Figure 3d). At 20 weeks, three‐way ANOVA revealed significant effects of stimulation intensity (*F*_[1.524,47.23]_ = 55.02, *p* < 0.0001), genotype (*F*_[1,31]_ = 57.27, *p* < 0.0001), and a significant genotype × stimulation intensity interaction (*F*_[5155]_ = 37.97, *p* < 0.0001) (Figure 3e). Simple main effects analysis indicated that dopamine release in C − k and C + k mice did not differ. However, dopamine release significantly differed between MP − k and MP + k groups (significant effect of diet (*F*_[1,15]_ = 18.74, *p* = 0.0006) and stimulation intensity × diet interaction (*F*_[5,75]_ = 19.43, *p* < 0.0001)) (Figure 3f). Post hoc tests indicated these effects were driven by significant or near‐significant increases in dopamine release in the MP + k mice at 600 (*p* = 0.0483) and 800 (*p* = 0.0579) μA. Greater statistical detail is available in the supplemental materials.

### 3.5 KEED does not increase cell survival, striatal TH+ axons or tissue dopamine content

**FIGURE 4:**
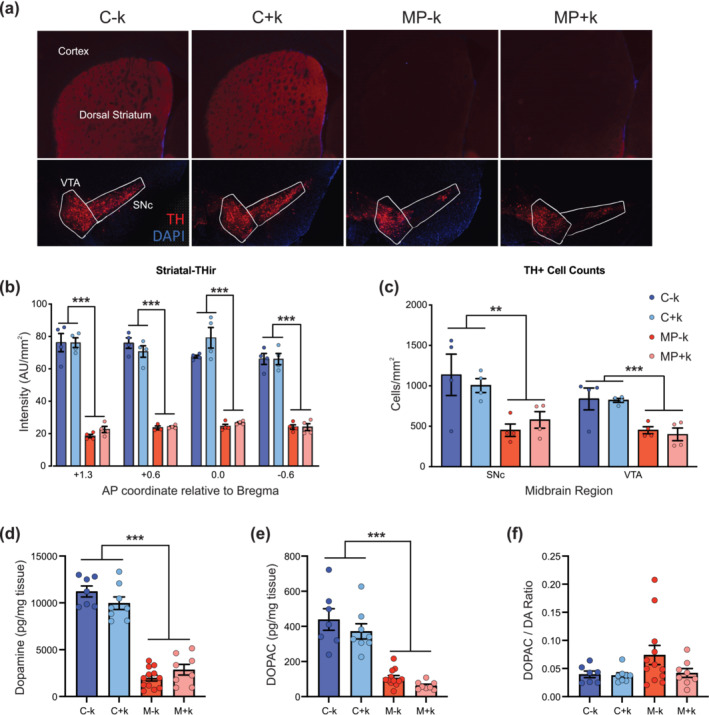
KEED does not preserve dopamine neurons or striatal axon loss in 20‐week‐old MitoPark mice. (a) Representative immunohistochemistry images of dorsal striatum (upper row) and midbrain dopamine neurons (lower row). (b) Striatal tyrosine hydroxylase‐immunoreactivity and midbrain dopamine neurons (c) were decreased in both MitoPark groups. Dopamine (d) and DOPAC (e) content were decreased in dorsal striatal tissue from 20‐week‐old MitoPark mice relative to controls. DOPAC/dopamine ratios (f) did not differ significantly across groups. *n* = 4 mice per group for immunohistochemistry experiments and *n* = 7–12 mice per group for biochemistry experiments. **p < 0.01; ***p < 0.001. KEED, ketone ester–enriched diet.

To determine if the KEED amelioration of dopamine release deficits was due to preservation of dopamine neurons or axons, we performed immunohistochemistry for tyrosine hydroxylase (TH) (Figure 4a). Across the anterior–posterior gradient of the dorsal striatum, MP mice had significantly decreased TH+ immunoreactivity compared to controls (three‐way repeated measures mixed‐effects analysis, significant main effect of genotype, *F*_[1,12]_ = 435.0, *p* < 0.0001), indicating dopamine axons degenerated regardless of diet (Figure 4b). Similarly, TH+ cell counts in the SNc and VTA indicated a genotype‐ but not diet‐dependent decrease in cell count (three‐way repeated measures ANOVA, significant main effect of genotype, *F*_[1,12]_ = 19.60, *p* = 0.0008) (Figure 4c). GC–MS analyses of flash‐frozen striatal tissue indicated that, although dopamine and DOPAC content was significantly diminished in MP mice (two‐way ANOVAs, dopamine: significant main effect of genotype, *F*_(1,31)_ = 245.1, *p* < 0.0001; DOPAC: significant main effect of genotype, *F*_(1,31)_ = 81.38, *p* < 0.0001), the KEED did not increase content of either compound (Figure 4d,e). Sidak's multiple comparison tests confirmed that MP mice, regardless of diet, had similar dopamine and DOPAC levels. Groups did not differ in DOPAC/DA ratio, which is thought to be a correlate of monoamine oxidase (MAO) activity and levels of oxidative stress. (Spina & Cohen, 1989) Thus, despite the beneficial KEED effects on motor deficits and dopamine release, the diet does not seem to slow neurodegeneration or increase striatal dopamine levels.

### 3.6 Late KEED intervention delays but does not rescue motor deficits

**FIGURE 5:**
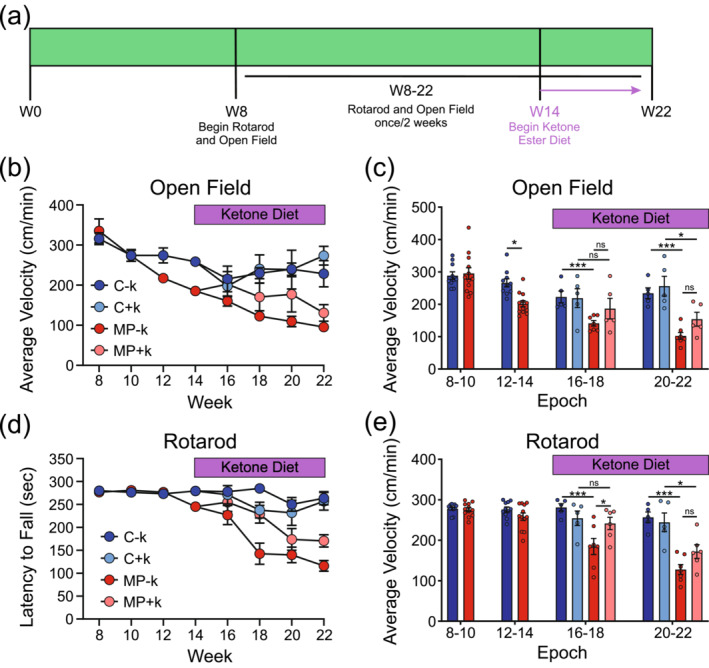
Beginning ketone ester enriched diet after motor deficit onset may slow progressive motor dysfunction in MitoPark mice. (a) Experimental timeline illustrating behavioral testing and KEED onset. (b, c) MitoPark mice fed either diet exhibited decreased locomotion in the open field relative to control groups. (d, e) MitoPark mice fed the control diet performed progressively worse on the rotarod across the study (d). MitoPark mice fed the KEED had improved motor function initially but failed to maintain this improvement. *n* = 5–7 mice per group. *p < 0.05; ***p < 0.001. KEED, ketone ester–enriched diet.

Our initial experiment prevented the development of motor deficits in MP mice up to 20 weeks. However, we began the KEED at 4 weeks, before any motor deficits emerged. This schedule is not comparable to treatment of PD in humans, as treatments begin after symptom onset. Thus, we tested whether the KEED would benefit subjects after the onset of motor deficits. Mice began rotarod and open field testing at 8 weeks and KEED began at 14 weeks (after onset of both open field and rotarod deficits in MP − k mice; Figure 2). The endpoint was extended to 22 weeks to better capture the potential effects of KEED intervention (Figure 5a). Behavioural data were binned into epochs as before (8–10, 12–14, 16–18 and 20–22 weeks).

Before KEED intervention (first two epochs) in the open field, we found a significant effect of time (two‐way RM ANOVA, *F*_[1,20]_ = 47.95, *p* < 0.0001) and a significant time × genotype interaction (*F*_[1,20]_ = 18.12, *p* = 0.0004), but no effect of genotype alone. Sidak's multiple comparisons tests indicated that MP, but not control, mice decreased movement from early to mid‐time points (*p* < 0.0001) and that at the mid time point, MP mice moved significantly less than controls (*p* = 0.0031). After dietary intervention (final two epochs), a significant effect of genotype (three‐way RM ANOVA, *F*_(1,18)_ = 20.24, *p* = 0.0003), and time × genotype interaction (*F*_(1,18)_ = 14.03, *p* = 0.0015) were observed. All other effects and interactions did not reach statistical significance (*p* > 0.05). To further investigate the effects that reached significance, we analyzed the data across genotype and diet factors with two‐way RM ANOVAs.

**Genotype**: For SD mice (C − k and MP − k), there were genotype (*F*_[1,10]_ = 38.68, *p* < 0.0001) and time × genotype interaction (*F*_[1,10]_ = 12.94, *p* = 0.0049) effects. Sidak's multiple comparison tests revealed that this effect was due to MP − k mice moving less over time (*p* = 0.0031), and MP mice moving less at both postdiet time points (16–18: *p* = 0.0006; 20–22: *p* < 0.0001). For mice on the KEED (C + k and MP + k), neither effect (Genotype and Time) or their interaction reached significance, indicating C + k and MP + k mice did not differ after dietary intervention; however, the lack of a diet effect or diet‐related interactions in the three‐way ANOVA obfuscates the meaning of this lack of statistical significance.

**Diet**: For control mice (C − k and C + k), neither effect (Time and Diet) nor their interaction reached statistical significance. For MP mice (MP − k and MP + k), there was a significant effect of time (*F*_[1,10]_ = 14.68, *p* = 0.0033). Post hoc tests indicated that only MP − k mice deteriorated over time (MP − k: *p* = 0.0180), but MP groups did not differ from each other at either time point. Overall, these data suggest a potentially subtle effect of late diet intervention on delaying spontaneous movement deficits.

On the rotarod, before KEED intervention, there were no significant effects or interactions, although the effect of epoch was near significance (two‐way RM ANOVA, epoch: *F*_[1,23]_ = 3.939, *p* = 0.0592). Sidak's multiple comparison tests revealed that although there were no differences between genotypes at either time point (*p* > 0.05), only MP mice differed in time spent on rotarod between early and mid‐time points (Control: *p* = 0.9600; MP: *p* = 0.0252). After intervention, a three‐way RM ANOVA indicated significant effects of time (*F*_[1,19]_ = 15.70, *p* = 0.0008) and genotype (*F*_[1,19]_ = 33.84, *p* < 0.0001), along with significant time × genotype (*F*_[1,19]_ = 5.121, *p* = 0.0356) and genotype × diet (*F*_[1,19]_ = 6.916, *p* = 0.0165) interactions. As before, we analyzed the data in separated two‐way ANOVAs to characterize the significant effects and interactions.

**Genotype**: For SD mice (C − k and MP − k), there were main effects of time (*F*_[1,10]_ = 8.994, *p* = 0.0134) and genotype (*F*_[1,10]_ = 43.63, *p* < 0.0001), but no interactions. Sidak's post hoc tests suggest that only MP − k mice deteriorate in performance over time (C − k: *p* = 0.4593; MP − k: *p* = 0.0174), but MP − k mice perform worse than C − k mice at both time points (16–18: *p* = 0.0005; 20–22: *p* < 0.0001). For KEED mice (C + k and MP + k), there was an effect of time (*F*_[1,9]_ = 6.840, *p* = 0.0280), but no genotype effect or time × genotype interaction. Like SD mice, Sidak's tests indicated that C + k mice did not deteriorate over time (*p* = 0.8887), but MP + k mice did (*p* = 0.0159). Unlike SD mice, C + k and MP + k did not perform differently at the first time point post‐intervention, although MP + k mice eventually developed deficits relative to C + k mice (16–18: *p* = 0.8584; 20–22: *p* = 0.0231). These results suggest that late intervention may delay development of rotarod deficits.

**Diet**: Control mice (C − k and C + k) did not differ across time or diet (*p* > 0.05 for all). However, MP mice (MP − k and MP + k) displayed significant main effects of time (*F*_[1,11]_ = 19.22, *p* = 0.0011) and diet (*F*_[1,11]_ = 7.810, *p* = 0.0174), but no significant interaction (*p* > 0.05). Sidak's tests indicate these effects were driven by deterioration across time in both groups (MP − k: *p* = 0.0285; MP + k: *p* = 0.0145), and MP + k mice performing better than MP − k mice immediately after KEED onset (16–18: *p* = 0.0440) but equalled MP − k performance later (20–22: *p* = 0.1326). These analyses provide further evidence that the KEED may delay rotarod motor deficits.

## 4 DISCUSSION

KEEDs have been found to have beneficial effects for several conditions including diabetes (Jensen et al., 2020; Soto‐Mota et al., 2021), epilepsy (Ciarlone et al., 2016; Viggiano et al., 2015), Alzheimer's disease (Pawlosky et al., 2020), heart disease (Yurista et al., 2021) and radiation damage (Kemper et al., 2016). Here, we demonstrate that a KEED could also preserve motor function in a PD model. The MP model is notable for its progressive onset of motor deficits and dynamic response to L‐DOPA treatment (Ekstrand et al., 2007; Galter et al., 2010), mirroring the progression of motor symptoms in human PD patients (Carrarini et al., 2019; Ekstrand et al., 2007). Consistent with previous studies, we found deficits in spontaneous (open field) and forced locomotion (rotarod) (Chen et al., 2019) as well as dopamine neuron and striatal axon degeneration in MP mice. However, MP mice fed the KEED prior to motor deficit onset did not develop motor deficits through 20 weeks and had significantly increased dopamine release relative to MP − k controls. Notably, the preservation of dopamine was limited to evoked dopamine release as MP + k mice did not differ from MP − k mice in tonic tissue levels of striatal dopamine. In MP mice fed the KEED after the onset of motor deficits, there was no rescue of motor function; however, subsequent motor function degradation was delayed relative to MP − k mice. It is possible that a longer KEED treatment, or a higher circulating blood ketone level, would be of greater benefit. Under normal conditions, the brain relies primarily on glucose metabolism as its primary energy source. However, variations in the brain's energy demands may necessitate alternative fuel sources. For example, during periods of low glucose utilization or prolonged fasting, metabolism shifts to hepatic catabolism of triglycerides into long chain fatty acids (Krebs et al., 1971). However, because albumin‐bound fatty acid chains are not permeable to the blood brain barrier, excess acetyl‐CoA from β‐oxidation is converted into ketone bodies, including acetoacetate, acetone and D‐β‐hydroxybutyrate. Recently, administration of exogenous D‐BHB was shown to increase circulating ketone levels (Clarke et al., 2012; Myette‐Cote et al., 2018; Stubbs et al., 2018). D‐BHB crosses the blood–brain barrier, cell membrane and mitochondrial membrane using monocarboxylate transporters (Pierre & Pellerin, 2005), accumulating in the mitochondria, where it can be metabolized into acetyl‐CoA through 3‐hydroxybutyrate dehydrogenase 1 (Tieu et al., 2003). In this study, we utilized a D‐BHB KEED (Kashiwaya et al., 2013) to increase circulating blood ketone levels (Figure 1a).

We hypothesized that the KEED‐mediated amelioration of PD‐like motor deficits we observed may occur through two complementary biochemical mechanisms. Firstly, administration of D‐BHB increases the availability of NADPH (Pawlosky et al., 2017) by increasing mitochondrial acetyl‐CoA levels, which increases flux through the TCA cycle and increases citrate levels (Sato et al., 1995; Suissa et al., 2021). The increased citrate is oxidized to α‐ketoglutarate in the mitochondria, while NADP+ is reduced to NADPH (Han et al., 2017) which contributes to the redox control of the dihydrobiopterin‐tetrahydrobiopterin (BH2/BH4) couple. BH4 is a cofactor for TH (Homma et al., 2013), and increasing its bioavailability would enhance dopamine synthesis. Notably, PD patients have been shown to have reduced BH4 availability (Lovenberg et al., 1979). Thus, increased BH4 levels may contribute to the ketone‐mediated reduction of PD‐like motor symptoms by increasing dopamine synthesis. However, although evoked levels of dopamine were elevated in MP + k compared to MP − k, tonic striatal dopamine tissue levels did not differ significantly between groups, raising the possibility that the beneficial effect of the KEED is via enhanced vesicular packaging of dopamine. One possible mechanism supporting this would be through facilitation of VMAT2 activity, which is an active transporter, and D‐BHB maintenance of ATP levels under hypoglycaemic and increased mitochondrial dysfunction conditions (Haces et al., 2008). Further work is required to test these hypotheses. Nonetheless, the modest increase in dopamine release in MP + k mice may be sufficient to maintain normal motor function given the compensatory upregulation of dopamine receptors observed in PD patients (Kaasinen et al., 2021).

Dopaminergic neuron degeneration is a hallmark of PD. Contributing to this degeneration is oxidative stress and the decreased neuronal capacity to deal with reactive oxygen species (ROS), which at high levels can activate apoptosis/autophagy pathways (Filograna et al., 2016; Redza‐Dutordoir & Averill‐Bates, 2016). However, we found that neither striatal terminals nor midbrain cell bodies (as assessed by TH staining) were preserved in MP + k groups, suggesting that the diet did not affect neurodegeneration, though additional assessments would be required to state this definitively. Nonetheless, even though D‐BHB may play a role in quenching ROS, this effect did not translate into whole cell neuroprotective effects.

An additional mechanism through which the KEED may benefit PD symptoms is by alleviating cellular respiration deficits that are prevalent in PD (Henchcliffe & Beal, 2008) and a hallmark of the MP model (Ekstrand et al., 2007; Ricke et al., 2020). In vitro, D‐BHB promotes increased ATP production (Haces et al., 2008; Sato et al., 1995; Tieu et al., 2003). Furthermore, electron transport chain complex I dysfunction has been found to be a key driver of Parkinson's pathogenesis (Hattori et al., 1991; Mizuno et al., 1989; Schapira et al., 1989, 1990) and many complex I inhibitors, such as 6‐OHDA, MPTP, paraquat and rotenone, cause dopaminergic degeneration and Parkinsonian symptoms (Marella et al., 2009). The catabolism of D‐BHB increases succinate (Suissa et al., 2021), a substrate for complex II, and thus may circumvent complex I dysfunction.

In addition to this study, several other studies have investigated the effects of other treatments in MP mice. Langley et al. (2017) demonstrated the ability of a novel mitochondria‐targeted antioxidant, Mito‐Apocynin, to alleviate mitochondrial dysfunction, neurodegeneration and behavioural deficits in MP mice (Langley et al., 2017). Similarly, a recent study demonstrated the ability of quercetin, a plant flavonoid, to alleviate these deficits by improving mitochondrial bioenergetics (Ay et al., 2017). Furthermore, exercise has been shown to improve motor function in MP mice due to increased aerobic respiration (Lai et al., 2019). These results, combined with those of the present study, indicate that restoring mitochondrial function may be a promising therapeutic intervention for PD patients. However, it is worth noting that the MP model is specifically engineered to induce mitochondrial dysfunction (Ekstrand et al., 2007; Ricke et al., 2020) and future studies should confirm the efficacy of mitochondrial‐based interventions in other progressive models of PD, such as the α‐synuclein overexpression model (Lin et al., 2012). An additional consideration is the timing of KEED onset. For example, in our first experiment, the KEED was started when mice were 6 week old. This coincides with the onset of puberty for male mice and the end of puberty for female mice. During puberty and adolescence, the dopamine system is dynamic and it is possible that the KEED may impact its development (Reynolds & Flores, 2021). However, given the similar performance of control mice on either diet on the behavioural tasks, TH‐ir (in striatum and midbrain), and dopamine release, we do not believe that the KEED adversely affected the development of the dopamine system.

In summary, the present study establishes the potential for the use of ketone esters in the treatment of Parkinson's disease. MP mice fed a KEED had preserved motor function and striatal dopamine release relative to MP mice fed the SD. Although we were unable to determine a specific mechanism for this rescue, it is possible that by enhancing/preserving mitochondrial bioenergetics in MP mice fed the KEED, dopamine synthesis and vesicular packaging were preserved and accounts for the amelioration of motor deficits and dopamine release deficits. Future studies will assess this possibility in MP and other PD mouse models. Overall, our study demonstrates a potential beneficial effect of a ketone enriched diet on the progression of PD‐related symptomology.

## AUTHOR CONTRIBUTIONS

Designed research: MID, RLV, AGS, DML. Contributed essential reagents: MTK. Performed research: VRM, JAN, AGS, RJP. Analyzed data: JAN, AGS, RJP. Prepared manuscript: VRM, JAN, AGS, DML.

## CONFLICT OF INTEREST STATEMENT

RLV and MTK received royalties from patents owned by The National Institutes of Health, Oxford University, and TdeltaS Ltd., a University of Oxford company, established to commercialize the ketone ester. All other authors declare no financial interests or potential conflicts of interest.

### PEER REVIEW

The peer review history for this article is available at https://www.webofscience.com/api/gateway/wos/peer-review/10.1111/ejn.16601.

## DEDICATIONS

This paper is dedicated to the memory of Dr. Richard “Bud” Veech whose belief in the broad therapeutic benefits of a KEED inspired this study.

## Supporting information


**Data S1.** Supporting Information.

## Data Availability

The data that support the findings of this study are available at lsuhs.figshare.com or by request from the corresponding authors.
